# Circular RNA hsa_circ_0000317 inhibits non-small cell lung cancer progression through regulating microRNA-494-3p/phosphatase and tensin homolog deleted on chromosome 10 axis

**DOI:** 10.1016/j.clinsp.2022.100086

**Published:** 2022-07-30

**Authors:** Shihui Xia, Zengwang Zhang

**Affiliations:** Department of Cardiothoracic Surgery, Xiangyang Central Hospital, Affiliated Hospital of Hubei University of Arts and Science, Hubei, China

**Keywords:** Circ_0000317, miR-494-3p, PTEN, Non-small cell lung cancer cells

## Abstract

**Background:**

Circular RNA (circRNA), a group of non-coding RNA, is pivotal in the progression of various cancers, including Non-Small Cell Lung Cancer (NSCLC). Some circRNAs have been reported to be implicated in the progression of NSCLC, however, the function and molecular mechanism of hsa_circ_0000317 (circ_0000317) in NSCLC have not been fully understood.

**Methods:**

The significantly differentially expressed circRNA in NSCLC tissues, circ_0000317, was screened out by microarray. Circ_0000317, microRNA(miR)-494-3p and Phosphatase and Tensin Homolog Deleted on Chromosome 10 (PTEN) expressions in NSCLC tissues were respectively probed by quantitative real-time polymerase chain reaction and western blot assay. MTT and Transwell assays were adopted to examine the growth, migration, and invasion of NSCLC cells. Bioinformatics, luciferase reporter gene assay, RNA immunoprecipitation, and RNA pull-down assay were conducted to probe the relationships among circ_0000317, miR-494-3p, and PTEN.

**Results:**

Circ_0000317 expression level was reduced in NSCLC tissues and cell lines. Circ_0000317 expression in NSCLC patients was associated with TNM stage and lymphatic metastasis. Circ_0000317 overexpression restrained the proliferation, migration, and invasion of NSCLC cells, but co-transfection of miR-494-3p mimics partially reversed this effect. In addition, circ_0000317, was identified as a competitive endogenous RNA, which could sponge miR-494-3p to increase PTEN expression and activate PI3K/AKT pathway.

**Conclusion:**

Circ_0000317, inhibits NSCLC progression via modulating miR-494-3p/PTEN/PI3K/AKT pathway.

## Introduction

Lung cancer is not only the most frequently diagnosed cancer but also the leading cause of cancer-related death.^1^^,^^2^ Non-Small Cell Lung Cancer (NSCLC) accounts for approximately 80%‒85% of all lung cancer cases, and the 5-year rate for patients with metastatic NSCLC is less than 5%.^3^, ^4^, ^5^, ^6^ Most NSCLC patients have entered the advanced stage when they are diagnosed, and the prognosis of the patients with recurrence and distant metastasis is extremely poor.^6^ It is critical to find new therapeutic targets for further improving NSCLC treatments.

Circular RNAs (circRNAs), existing stably in tissues and cells, are structurally characterized by closed loops with neither 5′ cap nor 3′ polyadenylated tail.^7^^,^^8^ As reported, circRNAs, as vital regulators of gene expression, are differentially expressed in tumor tissues and cell lines. They participate in cancer progression and are closely related to the disease status and prognosis of the patients.^9^ CircRNAs can decoy microRNA (miRNA), to reduce the abundance of miRNAs in the cytoplasm.^10^ This circRNA-miRNA regulatory network acts on the target genes which are involved in the biological processes of cancer cells.^11^ The research on the biological function of circRNAs in NSCLC is helpful to offer new ideas for the diagnosis and treatment of NSCLC.

MiRNAs are short endogenous non-coding RNA with a length of 20‒22*nt*, which can partake in regulating physiological and pathological processes.^12^, ^13^, ^14^ Abnormal expression of miRNAs contributes to tumorigenesis.^15^ MiR-494-3p expression is associated with the poor survival time of lung cancer patients; besides, miR-494-3p improves lung cancer cell viability and metastasis.^16^ However, the detailed molecular mechanism of miR-494-3p in promoting the progression of NSCLC has not been fully clarified.

In this work, the authors adopted circRNA microarray analysis to screen out the differentially expressed circRNAs in NSCLC tissues. The authors identified a circRNA, hsa_circ_0000317, which was remarkedly decreased in NSCLC tissue samples. Functionally, silencing circ_0000317 could strengthen the malignant biological behaviors of NSCLC cells. Mechanistically, circ_0000317 could adsorb miR-494-3p to up-regulate Phosphatase and Tensin Homolog Deleted on Chromosome 10 (PTEN), thus inhibiting the progression of NSCLC.

## Material and methods

### Patient tissues and cell culture

Sixty-seven NSCLC tissues and adjacent normal lung tissues were available from 67 NSCLC patients from Xiangyang Central Hospital, Affiliated Hospital of Hubei University of Arts and Sciences. Fresh tumor tissues were confirmed by pathological examination, frozen in liquid nitrogen and immediately stored at -80°C. This work, with patients’ written informed consent, was endorsed by the Ethics Committee of Xiangyang Central Hospital, Affiliated Hospital of Hubei University of Arts and Sciences. All procedures were performed according to the guidelines of the Declaration of Helsinki. The immortalized human bronchial epithelium cells BEAS-2B, human NSCLC cell lines (A549, H460, PC9, H1299, and SPC-A1), and Human Embryonic Kidney cell line (HEK293T) were from the Type Culture Collection of the Chinese Academy of Sciences (Shanghai, China) and subsequently cultured in RPMI 1640 medium (Gibco, Carlsbad, CA, USA) with 10% fetal bovine serum (FBS, Gibco, Carlsbad, CA, USA), 100 μg/mL streptomycin and 100 U/mL penicillin in an incubator in 5% CO_2_ at 37°C.

### Cell transfection

Circ_0000317 overexpressing vector pcDNA3.1-circ_0000317 and the empty vector pcDNA3.1, Small Interference RNA (siRNA) targeting circ_0000317 (si-circ_0000317#1, si-circ_0000317#2 and si-circ_0000317#3), miR-494-3p mimics and corresponding negative control (si-NC and miR-control) were available from GenePharma (Shanghai, China). The transfection was conducted with Lipofectamine® 2000 (Invitrogen, Carlsbad, CA, USA). 48h after the transfection, the cells were collected for detecting the transfection efficiency by quantitative Real-Time Polymerase Chain Reaction (qRT-PCR) and for subsequent experiments.

### RNA isolation and qRT-PCR

Total RNA was isolated by a RNAiso Plus kit (Takara, Dalian, China). cDNA was synthesized respectively by a PrimeScript RT Reagent Kit (Takara, Dalian, China) and a microRNA First-Strand cDNA Synthesis Kit (Sangon Biotech, Shanghai, China). qRT-PCR was conducted on an ABI PRISM® 7300 Sequence Detection System (Applied Biosystems, Carlsbad, CA, USA). The gene expressions were normalized by β-actin/U6 expression. All primer sequences were accordingly synthesized by RiboBio Co., Ltd. (Guangzhou, China), and the sequences of the primers are completely detailed in Table 1.

**Table 1:** Primer sequence.

Gene	Sequence
circ_0000317	F: 5’-GTGATCTGAAAGGGCCAGAG-3’
	R: 5’-TCCACATCACCCTTCACCTT-3’
PTEN	F: 5’-TAGAGCGTGCAGATAATGACAAGGA-3’
	R: 5’-TGAACTGCTAGCCTCTGGATTTGA-3’
β-actin	F: 5’-GGGAAATCGTGCGTGACATTAAG-3’
	R: 5’-TGTGTTGGCGTACAGGTCTTTG-3’
U6	F: 5’-ATTGGAACGATACAGAGAAGATT-3’
	R: 5’-GGAACGCTTCACGAATTTG-3’

F, Forward; R, Reverse.

### MTT assay

3 × 10^3^ cells per well were inoculated in 96-well plates. At different time points, 20 μL of MTT solution (5 mg/mL; Sigma-Aldrich, St. Louis, Mo, USA) was added to the cells and subsequently incubated for 4h. Then 200 μL of dimethyl sulfoxide (DMSO, Sigma-Aldrich, St. Louis, MO, USA) was loaded, and then the formazan was dissolved, and the absorbance at 490 nm was recorded by a microplate reader (Bio Tek Instruments, Inc., Winooski, VT, USA).

### Migration and invasion assays

Transfected cells were suspended in 200 μL of serum-free medium (2 × 10^4^ cells) and inoculated into the upper chambers of Transwell inserts (8 μm pore size, Costar, Cambridge, MA, USA), which were specifically coated with or without Matrigel (BD Biosciences, San Jose, CA, USA) for the invasion and migration assay, respectively. Medium containing 10% FBS was loaded into the bottom chamber. Subsequently, the cells were cultured at 37°C in 5% CO_2_ for 48h for the invasion assay and 24h for the migration assay. After that, the cells in the top chamber were wiped off with cotton swabs, and the cells on the lower surface were fixed with methanol, subsequently stained with 0.1% crystal violet, and ultimately photographed under a microscope (Olympus, Japan).

### Fluorescence in situ hybridization (FISH)

The subcellular localization of hsa_circ_0000317 in NSCLC was identified by a FISH kit (Gefanbio, Shanghai, China). Briefly, A549 and PC9 cells were seeded on slides which were placed in dishes and cultured until 70%–80% confluence. Then, the cells were fixed in 4% paraformaldehyde for 10 min at room temperature and treated with protease K. Then, the cells were incubated with a FITC-labeled circ_0000317 probe at 65°C for 48h. then the cell nuclei were stained with 4,6-Diamidino-2-Phenylindole (DAPI) (Invitrogen, Carlsbad, CA, USA) for 10 min in the dark. Next, the cells were observed using a fluorescence microscope (Leica Microsystems, Mannheim, Germany).

### Dual-luciferase reporter gene assay

The sequences of circ_0000317 and PTEN 3ʹUTR containing the Wilt Type (WT) or Mutated Type (MUT) predicted binding sites of miR-494-3p were subcloned into the pmirGLO Dual-Luciferase miRNA Target Expression Vector (Promega, Madison, WI, USA) to construct circ-WT and PTEN-WT, circ-MUT and PTEN-MUT reporter vectors. HEK293T cells were co-transfected with the reporter vectors and miR-494-3p mimics or miRNA control by Lipofectamine® 2000 (Invitrogen, Carlsbad, CA, USA). 24h later, the firefly and Renilla luciferase activity in each group was examined by the Dual-Luciferase Reporter Assay System (Promega, Madison, WI, USA). The relative firefly luciferase activity was normalized to Renilla luciferase activity.

### RNA immunoprecipitation (RIP) assay

RIP assay was conducted with an EZ-Magna RIP Kit (EMD Millipore, Billerica, MA, USA). NSCLC cells were lysed in RIP lysis buffer plus cocktail (Roche Diagnostics, Shanghai, China). The supernatants were subsequently incubated with magnetic beads conjugated with primary antibodies of human Immunoglobulin G (IgG) or Argonaute 2 (Ago2) (EMD Millipore, Billerica, MA, USA). Next, the proteins in the immunoprecipitate were then degraded with proteinase K, and then immunoprecipitated RNA was then extracted. At last, purified RNA was used for the qRT-PCR assay.

### RNA pull-down assay

NSCLC cells transfected with biotinylated miR-494-3p mimic-biotin (Bio-miR-494) and its Negative Control (Bio-NC) were harvested and lysed in lysis buffer (Ambion, Austin, Texas, USA). Next, the lysate was incubated with streptavidin magnetic beads. Next, the pulled-down RNA was extracted from the complex and analyzed by the qRT-PCR assay. miR-494-3p mimic-biotin (Bio-miR-494) and its Negative Control (Bio-NC) were designed by GenePharma (Shanghai, China).

### Western blot

Cells in different groups were lysed with RIPA lysis buffer (Beyotime, Shanghai, China), and protein was subsequently quantified by a bicinchoninic acid kit (Beyotime, Shanghai, China). Next, protein extractions were resolved by sodium dodecyl sulfate-polyacrylamide gel electrophoresis, and then the protein was accordingly transferred to polyvinylidene fluoride (PVDF) membranes (Sigma-Aldrich, St. Louis, MO, USA). Next, the membranes were blocked in 5% skim milk at room temperature for 1h. Then the membranes were incubated with anti-PTEN antibody (ab170941, 1:1000, Abcam, Cambridge, UK), Anti-Phospho-Phosphatidylinositol 3-Kinase (PI3K) pantibody (ab182651, 1:1000, Abcam, Cambridge, UK), anti-phospho-AKT antibody (ab81283, 1:1000, Abcam, Cambridge, UK), and anti-β-actin primary antibody (ab8226, 1:2000, Abcam, Cambridge, UK), respectively at 4°C overnight. The next day, the membranes were accordingly incubated with secondary antibody (1:5000, Abcam, Cambridge, UK). The protein immunoblots were subsequently visualized by the ECL Western blotting substrate (Promega, Madison, WI, USA) and then scanned by a Bio-Rad image analysis system (Bio-Rad, Hercules, VA, USA).

### Statistical analysis

All experiments were executed not less than three times. Statistical analysis was performed with SPSS v22.0 (SPSS, Inc, Chicago, IL, USA), with data presented as means ± standard deviation. Besides, student's *t*-test or one-way Analysis of Variance (ANOVA) with Tukey's post hoc test was, respectively, performed to compare two or multiple groups. A Chi-Square test was conducted to analyze the correlation between circ_0000317 expression and the clinical features. The correlation between the two clinical indicators was evaluated by Pearson's correlation analysis. Statistically, p < 0.05 is meaningful.

## Results

### Circ_0000317 is downregulated in NSCLC

**Fig. 1:**
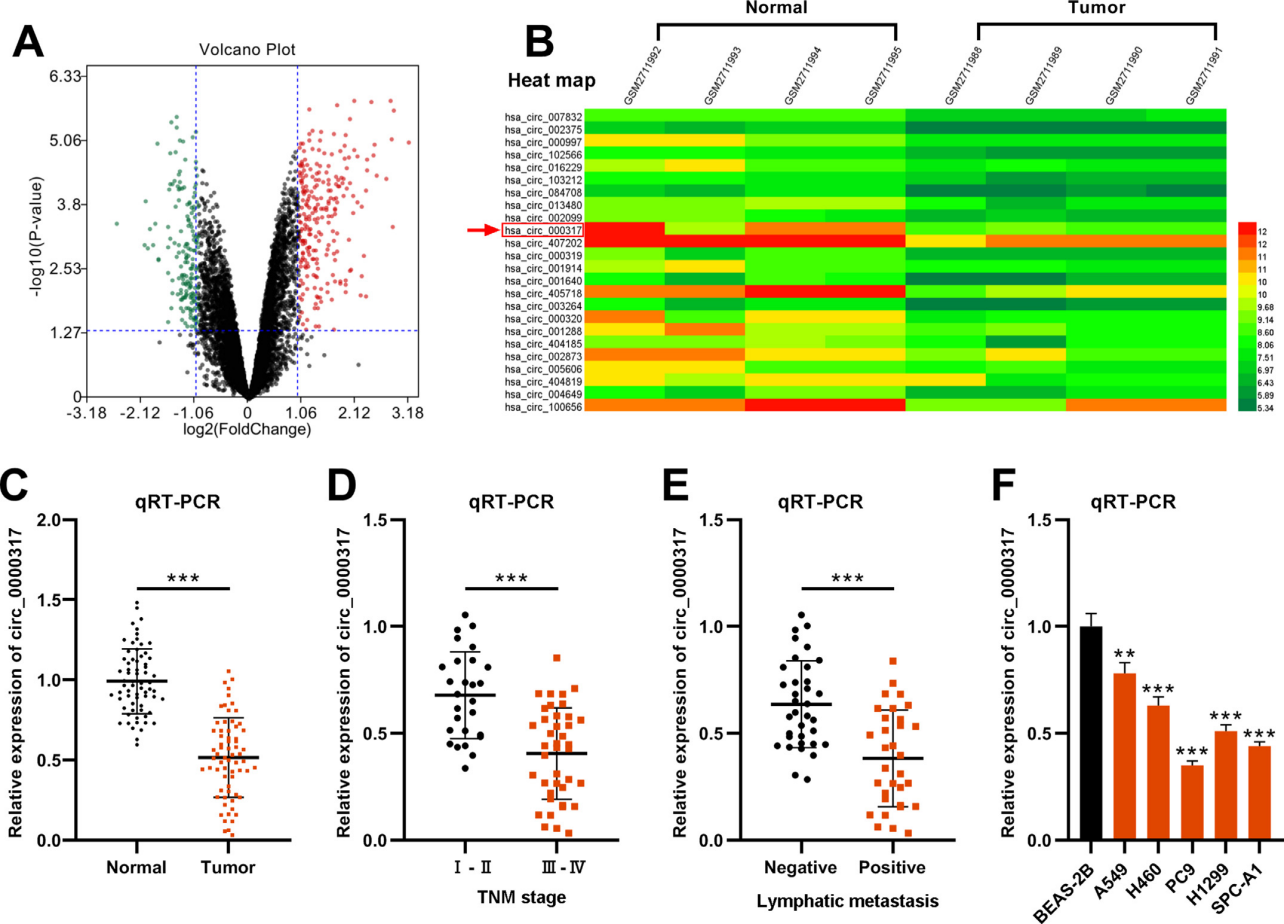
Circ_0000317 is downregulated in NSCLC tissues. (A) Volcano plot showed the differentially expressed circRNAs of lung adenocarcinoma tissues and matched non-tumor tissues in GSE101684. The cut-off criteria of values were as follows: ∣Log2(fold change)∣ > 1 and p < 0.05. The red points represented significantly up-regulated circRNAs, and the green points represented significantly down-regulated differentially expressed circRNAs, and the black points represented circRNAs with no statistically significant difference. (B) Heat map showed that 24 circRNAs were significantly differentially expressed in lung adenocarcinoma tissues. (C) The expression levels of circ_0000317 in 67 paired of NSCLC tissues (red points) and adjacent normal tissues (black points) were examined by qRT-PCR. (D) The expression levels of circ_0000317 in NSCLC tissues with lower TNM stage (black points, I‒II, n = 27) and higher stage (red points, III‒IV, n = 40) were examined by qRT-PCR. (E) The expression levels of circ_0000317 in NSCLC tissues with (red points, Positive, n = 32) and without (black points, Negative, n = 35) distant metastasis were analyzed by qRT-PCR. (F) The levels of circ_0000317 in NSCLC cell lines (A549, H460, PC9, H1299 and SPC-A1) and the immortalized Bronchial Epithelium Cell line (BEAS-2B) were examined by qRT-PCR. **p < 0.01, and ***p < 0.001.

First of all, the authors analyzed a circRNA expression profile GSE101684 downloaded from the Gene Expression Omnibus (GEO) database, which included the circRNA expression data of 4 pairs of lung adenocarcinoma tissues and matched non-tumor tissues, and discovered that there were 174 down-regulated circRNAs and 236 up-regulated circRNAs in lung adenocarcinoma tissues (p < 0.05, ∣Log2FC∣ > 1), (Fig. 1A). Circ_0000317 showed the most remarkable down-regulation in lung adenocarcinoma tissues [log2(Change fold) = -2.6111224, p = 0.000359], (Fig. 1B). Consistently, qRT-PCR revealed that circ_0000317 expression in NSCLC tissues was lower than that in normal tissues adjacent to cancer (Fig. 1C). Next, the 67 cases of NSCLC tissue samples were divided into circ_0000317 low expression group (n = 34) and circ_0000317 high expression group (n = 33). It was observed that the low expression of circ_0000317 was associated with the advanced TNM stage and lymphatic metastasis of NSCLC patients (Table 2, Fig. 1D and E). qRT-PCR also revealed that circ_0000317 expression in NSCLC cell lines (A549, H460, PC9, H1299 and SPC-A1) were significantly reduced as against that of BEAS-2B (Fig. 1F). All these data implied that circ_0000317 is lowly expressed in NSCLC and could probably work as a regulator in NSCLC progression.

**Table 2:** Correlations between circ_0000317 expression and clinical characteristics in NSCLC patients.

Pathological indicators	Number of patients	Circ_0000317		p-value (^a^p < 0.05)
		Low expression	High expression	
All cases	67	34	33	
**Age**				
< 60	30	16	14	0.703
≥ 60	37	18	19	
**Gender**				
Female	19	12	7	0.201
Male	48	22	26	
**TNM stage**				
I-II	27	9	18	0.019^a^
III-IV	40	25	15	
**Tumor size**				
< 4 cm	40	19	21	0.518
≥ 4 cm	27	15	12	
**Lymphatic metastasis**				
Negative	35	13	22	0.020*
Positive	32	21	11	
**Pathological type**				
Squamous cell carcinoma	59	30	29	0.964
Adenocarcinoma	8	4	4	
**Differentiation**				
Well + Moderate	40	24	16	0.065
Poor	27	10	17	

### Knocking down circ_0000317 promotes the growth, migration, and invasion of NSCLC

**Fig. 2:**
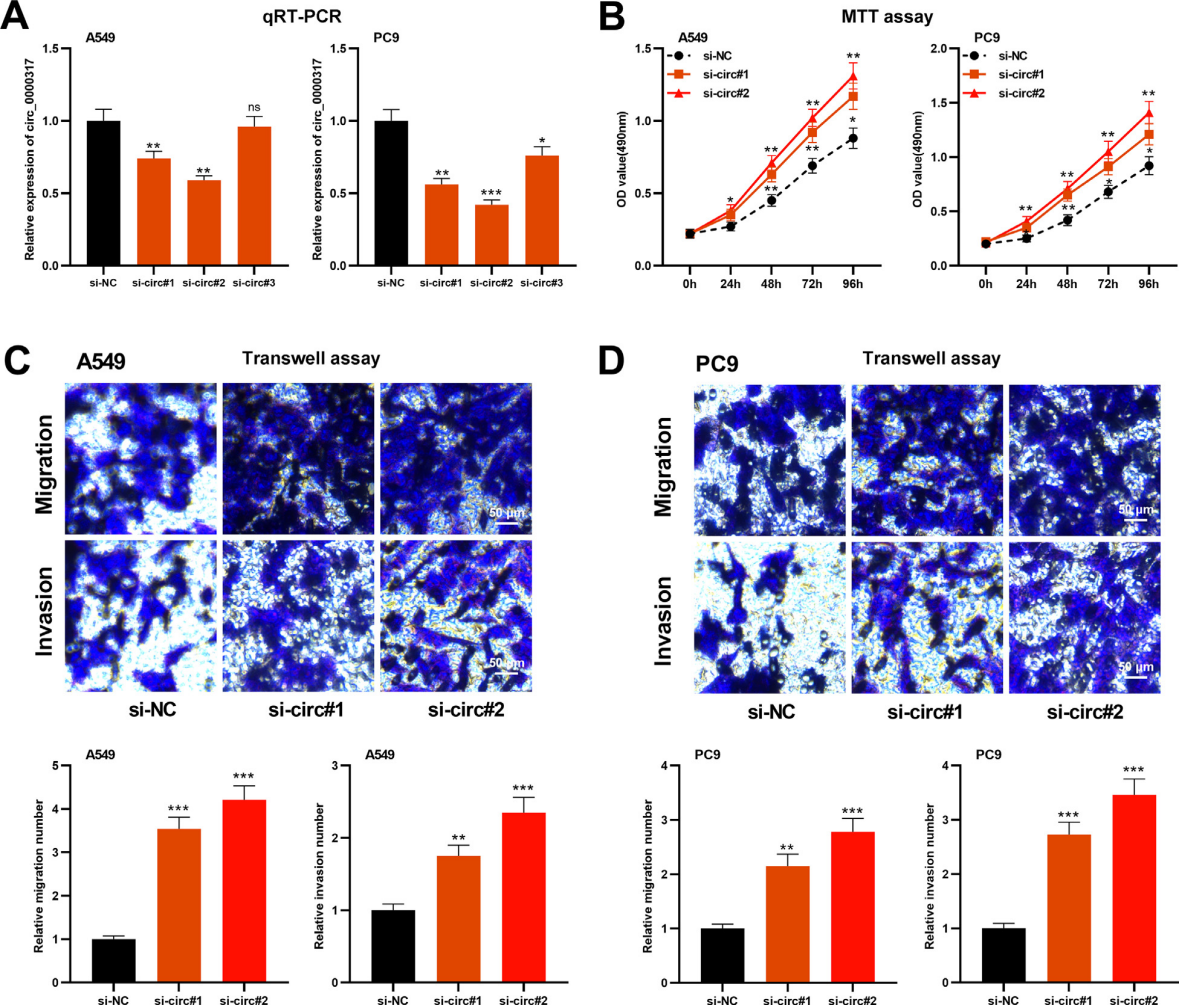
Knocking down circ_0000317 promotes the proliferation, migration, and invasion of NSCLC cells. (A) Si-NC, si-circ_0000317#1, si-circ_0000317#2 and si-circ_0000317#3 were transfected into A549 and PC9 cells, respectively, and the expression of circ_0000317 was detected by qRT-PCR. (B) MTT assay was used to detect the proliferation of A549 and PC9 cells transfected with si-circ_0000317#1 or si-circ_0000317#2. (C-D). Transwell assay was used to detect the migration and invasion of A549 and PC9 cells transfected with si-circ_0000317#1 or si-circ_0000317#2. si-NC, siRNA Negative Control; si-circ, siRNA targeting circ_0000317; ns, not statistically significant. *p < 0.05, **p < 0.01, and ***p < 0.001.

To delve into the role of circ_0000317 in NSCLC progression, A549 and PC9 cells were transfected with small interference RNA (siRNA) targeting circ_0000317 (si-circ_0000317#1, si-circ_0000317#2 and si-circ_0000317#3) and found that circ_0000317 in si-circ#1 and si-circ#2 showed higher knockdown efficiency (Fig. 2A). Therefore, si-circ#1 and si-circ#2 was chosen for the subsequent experiments. MTT assay and Transwell assay revealed that in comparison with the si-NC group, the cell viability, migration, and invasion of the NSCLC cells in the circ_0000317 knockdown group were significantly increased (Fig. 2B-D), implying that circ_0000317 was a potential tumor suppressor.

### Circ_0000317 sponges miR-494-3p in NSCLC cells

**Fig. 3:**
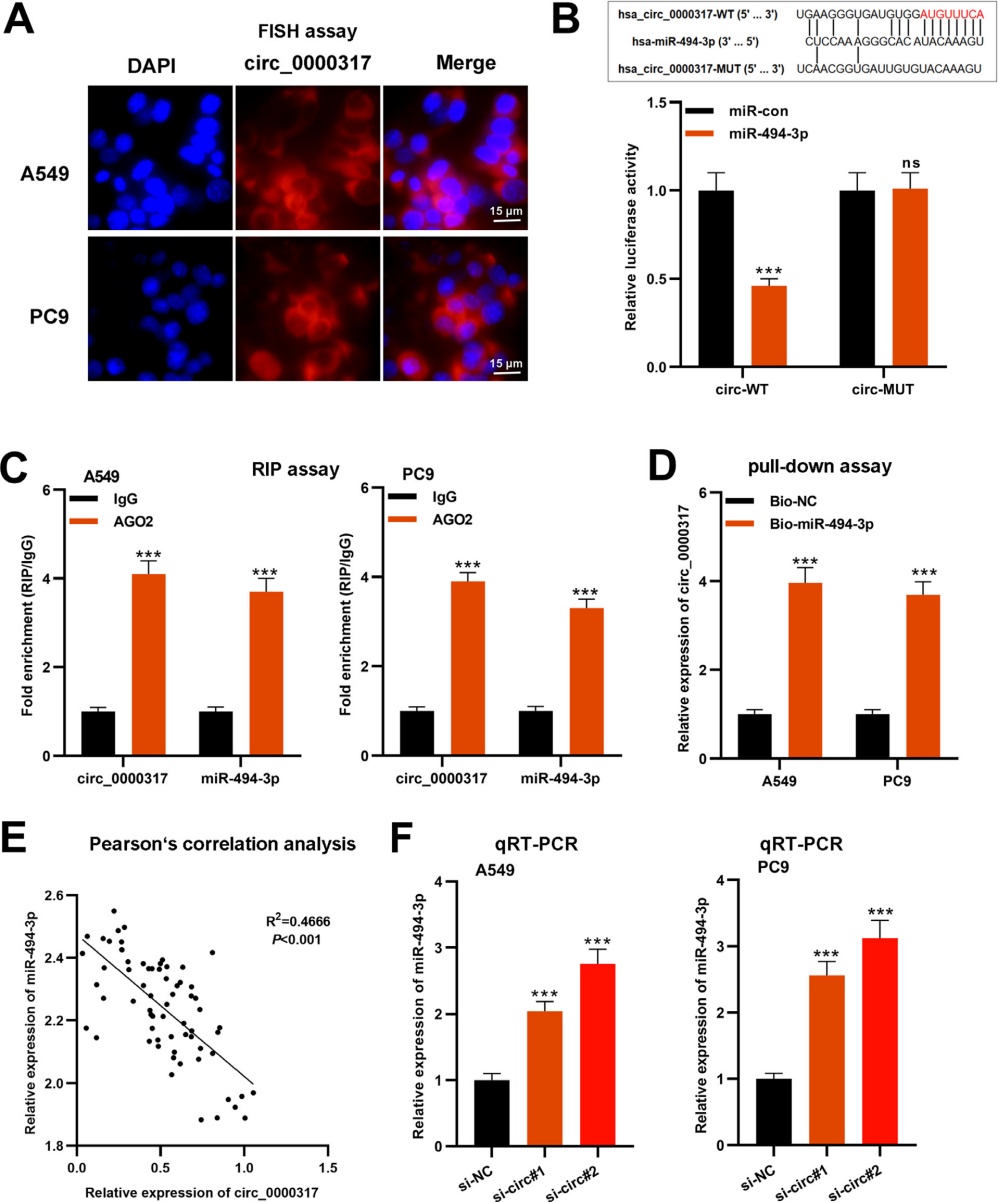
MiR-494-3p is the downstream target of circ_0000317. (A) FISH assays were performed to determine the localization of circ_0000317 in A549 and PC9 cells. (B) According to the binding site between circ_0000317 and miR-494-3p, circ_0000317-WT and circ_0000317-MUT luciferase reporter gene vectors were constructed. And miR-494-3p mimics or miR-control were co-transfected into HEK293T cells with circ_0000317-WT or circ_0000317-MUT. Then the luciferase activity of cells in each group was measured. (C-D) RIP assay (C) and RNA pull-down assay (D) were performed to verify the interaction of circ_0000317 and miR-494-3p. (E) Pearson's correlation analysis was employed to analyze the correlation between circ_0000317 expression and miR-494-3p expression in NSCLC tissues. (F) The expression of miR-494-3p in A549 and PC9 cells transfected with si-circ_0000317#1 and si-circ_0000317#2 was detected by qRT-PCR. Circ-WT, circ_0000317-WT; circ-MUT, circ_0000317-MUT; miR-con, miR-control; miR-494-3p, miR-494-3p mimic; ns, not statistically significant. ***p < 0.001.

To determine the molecular mechanism of circ_0000317 in NSCLC progression, first of all, the FISH assay was performed, and the results revealed that circ_0000317 was mainly distributed in the cytoplasm of A549 and PC9 cells (Fig. 3A). Then, the authors searched the CircInteractome database and noticed that miR-494-3p was a potential downstream target of circ_0000317 (Fig. 3B). Besides, dual-luciferase reporter gene assay highlighted that as against the control group, miR-494-3p mimic demonstrably restrained the luciferase activity of circ_0000317-WT reporter but did not significantly impact that of a circ_0000317-MUT reporter (Fig. 3B). RIP and pull-down assays further confirmed the direct interaction between circ_0000317 with miR-494-3p in NSCLC cells (Fig. 3C-D). Pearson's correlation analysis indicated that circ_0000317 expression was negatively correlated with miR-494-3p expression in NSCLC tissues (R^2^ = 0.4666, p < 0.001) (Fig. 3E). qRT-PCR revealed that miR-494-3p expression in A549 cells increased significantly after knocking down circ_0000317 (Fig. 3F). In a word, these experiments suggested that circ_0000317 targeted miR-494-3p and negatively modulated its expression in NSCLC cells.

### PTEN is the direct target of miR-494-3p in NSCLC

**Fig. 4:**
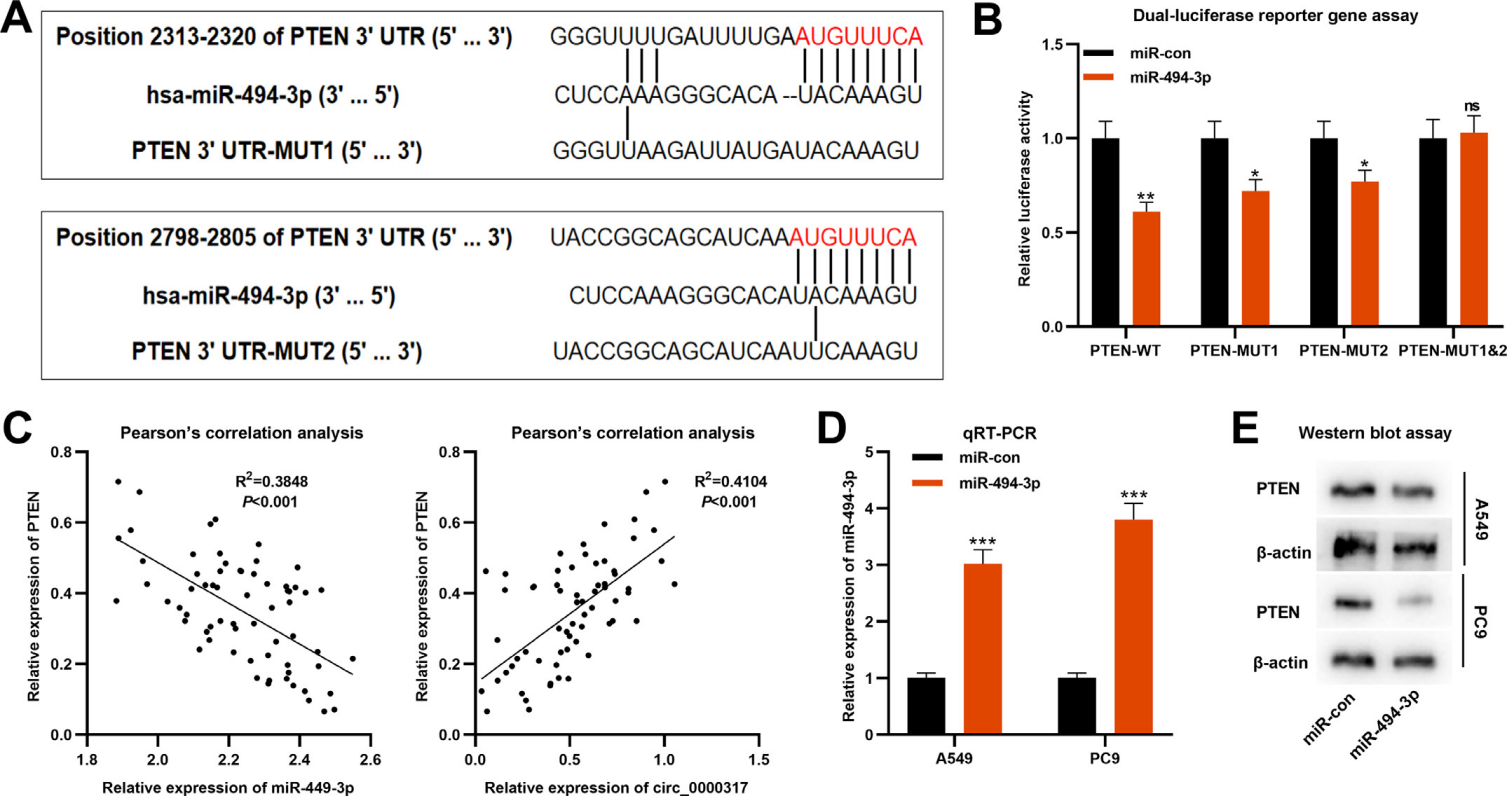
PTEN is the direct target of miR-494-3p. (A) According to the binding sites between PTEN 3′UTR and miR-494-3p, PTEN-WT, PTEN-MUT1, PTEN-MUT2 and PTEN-MUT1&2 luciferase reporter gene vectors were constructed. (B) MiR-494-3p mimic or miR-control were cotransfected into HEK293T cells with PTEN-WT or PTEN-MUT, respectively, and the luciferase activity of each group was determined. (C) Pearson's correlation analysis was employed to analyze the correlation between PTEN expression and miR-494-3p expression or circ_0000317 expression in NSCLC tissues. (D) The miR-494-3p mimic and miR-control were transfected into A549 and PC9 cells, and the expression of miR-494-3p was detected by qRT-PCR. (E) Western blot assay was used to detect the expression of PTEN in A549 and PC9 cells after overexpression of miR-494-3p. miR-con, miR-control; miR-494-3p, miR-494-3p mimic; ns, not statistically significant. *p < 0.05, **p < 0.01, and ***p < 0.001.

Next, the authors predicted that PTEN was a potential downstream target of miR-494-3p by TargetScan database, and there were two target binding sites in PTEN 3′UTR (Fig. 4A). Dual-luciferase reporter gene assay revealed that the luciferase activity of PTEN-WT, PTEN-MUT1 or PTEN-MUT2 luciferase reporters was significantly decreased by miR-494-3p mimics, while that of cells transfected with PTEN-MUT1&2 was not significantly affected (Fig. 4B). Pearson's correlation analysis confirmed that PTEN expression was negatively interrelated with miR-494-3p expression (R^2^ = 0.3848, p < 0.001), but positively correlated with circ_0000317 expression in NSCLC tissues (R^2^ = 0.4104, p < 0.001) (Fig. 4C). Besides, the miR-494-3p mimic was subsequently transfected into A549 cells to construct miR-494-3p overexpression model (Fig. 4D), and western blot assay proved that the expression level of PTEN protein in NSCLC cells was decreased significantly after up-regulating miR-494-3p (Fig. 4E). These findings revealed that PTEN was the direct target gene of miR-494-3p, and PTEN could be directly and negatively modulated by miR-494-3p.

### Circ_0000317 restrains the progression of NSCLC cells through miR-494-3p/PTEN axis

**Fig. 5:**
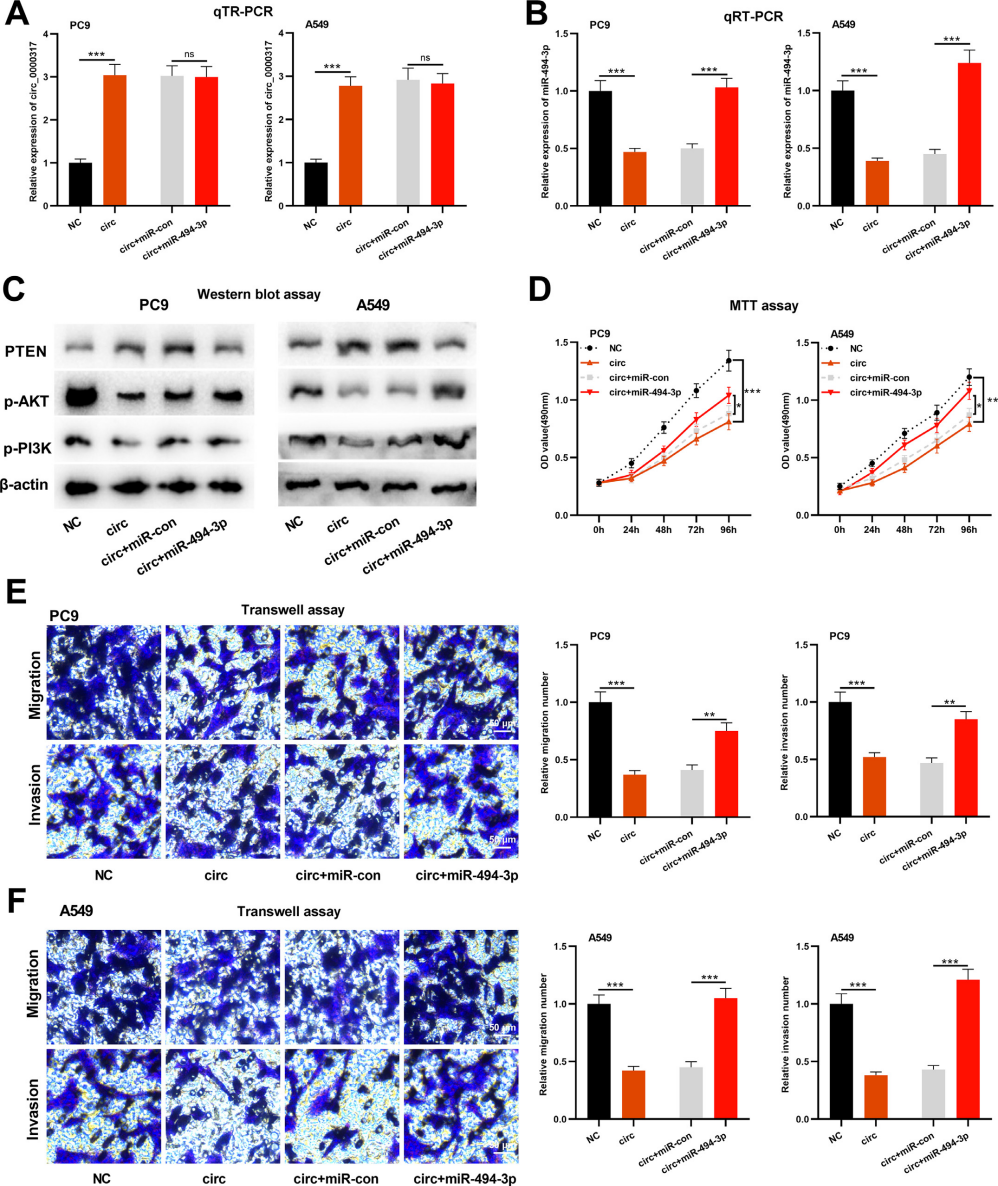
Circ_0000317 inhibits the progression of NSCLC through miR-494-3p/PTEN axis. PC9 and A549 cells were transfected with control vector, pcDNA3.1-circ_0000317, pcDNA3.1-circ_0000317 + miR-control, or pcDNA3.1-circ_0000317 + miR-494-3p mimics, respectively. (A) The expression of circ_0000317 in PC9 and A549 cells after transfection was detected by qRT-PCR. (B) The expression level of miR-494-3p in PC9 and A549 cells after transfection was detected by qRT-PCR. (C) Western blot assay was adopted to detect the protein expressions of PTEN, p-AKT, and p-PI3K in PC9 and A549 cells after transfection. (D) MTT assay was used to detect the proliferation of PC9 and A549 cells after transfection. (E‒F) Transwell assay was used to detect migration and invasion of PC9 and A549 cells after transfection. NC, Negative Control; circ, pcDNA3.1-circ_0000317; miR-con, miR-control; miR-494-3p, miR-494-3p mimic; ns, not statistically significant. * p < 0.05, **p < 0.01, and *** p < 0.001.

To further study whether circ_0000317 impacts NSCLC progression via modulating miR-494-3p/PTEN axis, the authors transfected pcDNA3.1-circ_0000317, pcDNA3.1-circ_0000317 + miR-control, and pcDNA3.1-circ_0000317 + miR-494-3p mimics in PC9 and A549 cells, respectively, followed by function compensation experiments (Fig. 5A-B). Western blot assay revealed that the protein expression of PTEN was raised in PC9 and A549 cells with circ_0000317 overexpression, while transfection of miR-494-3p mimic functioned oppositely; additionally, circ_0000317 overexpression suppressed the expressions of p-AKT and p-PI3K, but miR-494-3p mimic markedly promoted their expressions (Fig. 5C). MTT and Transwell assay confirmed that the growth, migration, and invasion of PC9 and A549 cells with circ_0000317 overexpression were decreased significantly, while transfection of miR-494-3p counteracted these effects (Fig. 5D-F). These findings highlighted that circ_0000317 could inhibit the progression of NSCLC cells via restraining miR-494-3p and the activation of PTEN/PI3K/AKT pathway.

## Discussion

More and more circRNAs have been confirmed to be implicated in the tumorigenesis, development, metastasis, and prognosis of NSCLC. Further study on the role of specific circRNAs in the development of NSCLC will help to find new targets for cancer treatments.^17^^,^^18^ Some circRNAs have been identified as crucial regulators in the progression of NSCLC. Specifically, it is reported that circ-ZKSCAN1 can inhibit the growth of NSCLC cells, and its high expression is closely related to the malignant characteristics of the tumor and the poor prognosis of the patients.^19^ CircRNA 100146 inhibits the proliferation and invasion of NSCLC cells and promotes apoptosis.^20^ Additionally, circ_0026134 enhances the viability and metastasis of NSCLC cells via targeting miR-1256 and miR-1287.^21^ In this study, through microarray analysis, the authors screened out a novel down-regulated circRNA in NSCLC tissue samples, namely, circ_0000317. The authors validated that circ_0000317 was remarkably low in NSCLC tissues and cells, and demonstrated it was associated with the adverse pathological characteristics of the patients. In addition, the growth, migration, and invasion ability of NSCLC cells with circ_0000317 silencing were enhanced. These findings suggest that circ_0000317 may impede the progression of NSCLC as a tumor suppressor.

Reportedly, circRNAs can exert their biological function via acting as miRNAs sponges, RNA-binding proteins, transcription regulators or protein translation templates.^22^^,^^23^ Among these mechanisms, the competitive endogenous RNA (ceRNA) mechanism is one of the hot spots in cancer research. For example, circ_0002483 inhibits NSCLC progression and enhanced the sensitivity of NSCLC cells to Taxol by sponging miR-182-5p.^24^ A study reports that circ_0078767 can restrain the proliferation, cycle progression, and invasion of NSCLC cells and promote apoptosis by inhibiting miR-330-3p and up-regulating RASSF1 expression.^25^ Also, circ-CMPK1/miR-302e/cyclin D1 axis is vital in regulating the cell cycle progression of NSCLC.^26^ In the present work, circ_0000317 was confirmed to be the molecular sponge of miR-494-3p. Reportedly, miR-494-3p, is implicated in the development of idiopathic pulmonary fibrosis.^27^ Besides, the level of circulating miR-494-3p has been reported to be associated with the overall survival time of lung cancer patients,^16^^,^^28^ and miR-494-3p is involved in regulating NSCLC cell proliferation, migration, invasion, and apoptosis.^16^^,^^29^ Intriguingly, the authors observed that PTEN, the famous tumor suppressor, was the downstream target of miR-494-3p. PTEN is a dual protein/lipid phosphatase, which can inhibit the progression of cancers through modulating PI3K/AKT pathway.^30^, ^31^, ^32^, ^33^ In NSCLC, the activation of PI3K/AKT pathway is associated with poor prognosis, which may be attributed to decreased PTEN level.^34^^,^^35^ In this study, the authors demonstrated that circ_0000317 negatively regulated miR-494-3p and positively regulated PTEN. In addition, miR-494-3p overexpression partially counteracted the promoting effect of circ_0000317 on PTEN expression, while the inhibitory effect of circ_0000317 on growth, migration, and invasion of NSCLC cells was weakened by the restoration of miR-494-3p. Meanwhile, circ_0000317 could suppress the activation of PTENPI3K/AKT pathway via miR-494-3p. Thereby, these data revealed that circ_0000317 may work as a ceRNA to inhibit NSCLC progression through miR-494-3p/PTEN/PI3K/AKT axis.

To recapitulate briefly, the circ_0000317 expression level declined in NSCLC tissues and cells, which is related to the malignant clinical features of NSCLC patients, and circ_0000317/miR-494-3p/PTEN/PI3K/AKT axis is implicated in the pathogenesis of NSCLC. The present data provide convincing evidence to support that circ_0000317 is a promising diagnostic biomarker and therapy target for NSCLC patients.

## Authors’ contributions

Conceived and designed the experiments: Shihui Xia and Zengwang Zhang; Performed the experiments: Shihui Xia; Analyzed the data: Shihui Xia and Zengwang Zhang; Wrote the paper: Shihui Xia and Zengwang Zhang.

All authors read and approved the final manuscript.

## Ethics statement

The present study was approved by the Ethics Review Board of Xiangyang Central Hospital, Affiliated Hospital of Hubei University of Arts and Sciences.

## Data availability statement

The data used to support the findings of this study are available from the corresponding author upon request.

## Declaration of Competing Interest

The authors declare no conflicts of interest.
